# Child Health Group

**Published:** 1963-04

**Authors:** 


### CHILD HEALTH GROUP, BRISTOL

At a meeting of the Group on 6th December 1962, Professor A. G. Watkins spoke of "Parent Counselling".

Family guidance during the illness of a child could be as important as the illness itself. Parents should be kept informed of their child's progress by repeated personal interviews. The doctor-parent relationship could affect the child's behaviour in hospital, on discharge, during convalescence, and in adjustment back to family life. A correct relationship was an important factor in parent education and in the prevention of subsequent illnesses. Yet this seemed to be a much neglected part of the average paediatrician's work in many parts of the world.

Four examples were given to illustrate the need for counselling:

(*a*) Mental defect, e.g. mongolism,

(*b*) Fatal illness, such as leukaemia or untreatable malignant disease,

(*c*) When recovery from an acute illness is expected, but some permanent disability is probable, e.g. rheumatic carditis, poliomyelitis,

(*d*) Chronic or recurring diseases, e.g. asthma, diabetes, nephrosis.

Each of these examples required a different approach in when, what, and how to tell the parents. The truth should be told, and it was the duty of the senior paediatrician and should not be left to the Junior resident.

The child was part of the family, and his illness was a family concern. The child was more important than his illness. If we were to retain our relationship with the family, through which child health must be propagated, then it was the task of those paediatricians whose experience fitted them for it to see the parents themselves. Without this, paediatric care was not complete.

